# Expansion of the MIRAgel Scleral Buckle With Findings Resembling Necrotizing Scleritis: A Case Report

**DOI:** 10.7759/cureus.73409

**Published:** 2024-11-10

**Authors:** Nanami Hata, Shinichiro Chujo, Hisashi Matsubara, Kumiko Kato, Mineo Kondo

**Affiliations:** 1 Department of Ophthalmology, Mie University Graduate School of Medicine, Tsu, JPN

**Keywords:** miragel, miragel removal, miragel scleral buckle, scleral buckle, uvetis

## Abstract

MIRAgel is a hydrogel buckle material that was widely used for scleral implantation surgery in the past. However, postoperative complications such as conjunctival hyperemia and impaired eye movements were reported due to postoperative expansion of the MIRAgel buckle. In addition, damage to the sclera due to hydrolysis of the MIRAgel buckle has been reported. Thus, surgical procedures had to be developed to remove the buckle. We report a case of an expansion of a MIRAgel buckle accompanied by scleral necrosis that resembled necrotizing scleritis. There are no studies on a safe method to remove a MIRAgel buckle when scleral melting is also present. We present our findings in a case with an expansion of the MIRAgel buckle accompanied by findings similar to necrotizing scleritis, where the buckle was safely removed using a suction tube that is frequently used in ophthalmic surgery.

## Introduction

MIRAgel is a hydrogel scleral buckle material that was widely used in scleral implantation surgery in the late 1980s [1]. The reason for its use was the belief that MIRAgel had a lower risk of postoperative infections than a silicone sponge [1]. However, there have been reports of late postoperative complications such as bulging of the conjunctiva due to an expansion of the MIRAgel, conjunctival hyperemia, and impaired eye movements [2-4]. The treatment for these complications required the removal of the MIRAgel buckles [5].

Forceps were initially used to remove the MIRAgel buckle, but it was reported that the removal was difficult because the inflated and deteriorated MIRAgel was easily fragmented. In addition, it has been reported the deterioration was accompanied by scleral erosion due to the hydrolysis of the MIRAgel [6].

There have also been reports of scleral perforation during the MIRAgel removal surgery [5]. In other words, the removal method using forceps that has been reported in the past may damage the uvea during removal in cases where scleral erosion is combined.

For these reasons, various methods have been used to remove the buckle safely without causing scleral perforation.

Most recently, Nakao et al. reported a method of using a surgical suction tube, a Yankauer suction catheter, to remove a deteriorated MIRAgel buckle [7]. It was possible to remove the buckle safely using this method, even though the diameter of the catheter was 4 mm. This catheter can be considered a surgical instrument, so there is still a possibility that there may be some difficulty in performing fine ophthalmic surgeries. However, this method may not be suitable for cases involving scleral erosion, as it requires fine manipulation. Therefore, it is necessary to devise an effective removal method for cases involving scleral erosion.

We report our findings in a case in which a degenerated MIRAgel buckle was safely removed using a Rosen suction tube (Daiichi Medical, Tokyo, Japan). In this eye, the degenerated MIRAgel buckle was associated with findings similar to necrotizing scleritis.

## Case presentation

An examination of a 49-year-old man at a neighborhood clinic found that the vision in his left eye was decreased, the conjunctiva was hyperemic, and the intraocular pressure (IOP) was 30 mmHg. It was initially believed that the cause of these changes was due to a bulging of the buckle, and he was followed by observation alone. His high IOP was controlled by topical medications, but in 2023, he was referred to our hospital because the vision in his left eye had worsened.

At our examination, he reported that he had undergone a retinal reattachment surgery for bilateral rhegmatogenous retinal detachment approximately 30 years earlier. His best-corrected visual acuity (BCVA) was 20/20 OD and 20/200 OS. His refractive error was approximately -7.0 diopters (D) in the left eye. The axial lengths were not measured.

**Figure 1:**
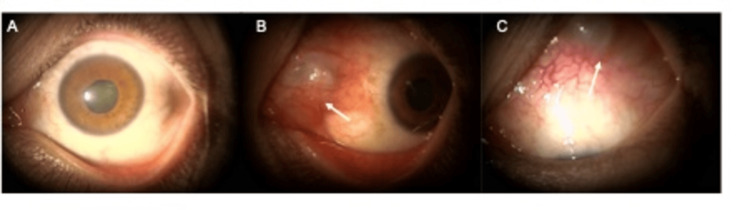
Slit-lamp images of the left eye of a patient who had an MIRAgel buckle implanted 30 years earlier for a retinal detachment. A: Frontal image. A mild cataract can be seen; B: A bulging of the MIRAgel buckle toward the nasal side can be seen; C: The MIRAgel buckle can be seen bulging on the superior surface of the eye.

The IOP was 13 mmHg OD and 8 mmHg OS. Slit-lamp examination showed no abnormal findings in the anterior chamber or the optic media of the right eye. However, a bulging of the conjunctiva, hyperemia, and moderate cataracts were detected in the left eye (Figure 1).

**Figure 2:**
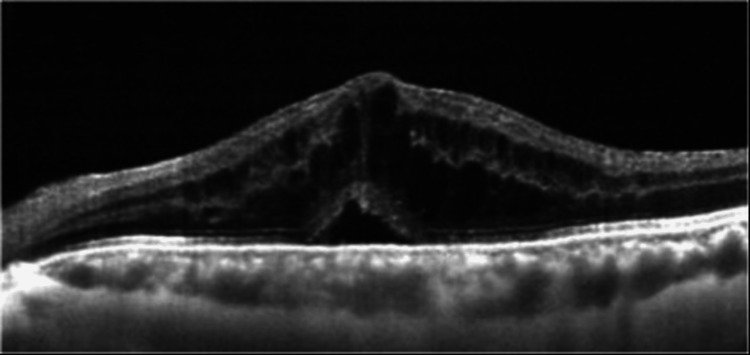
Optical coherence tomography (OCT) image of the left eye of this patient. The OCT image shows subretinal and intraretinal fluids.

The systemic blood tests were normal, and optical coherence tomography (OCT) showed macular edema in the left eye (Figure 2).

**Figure 3:**
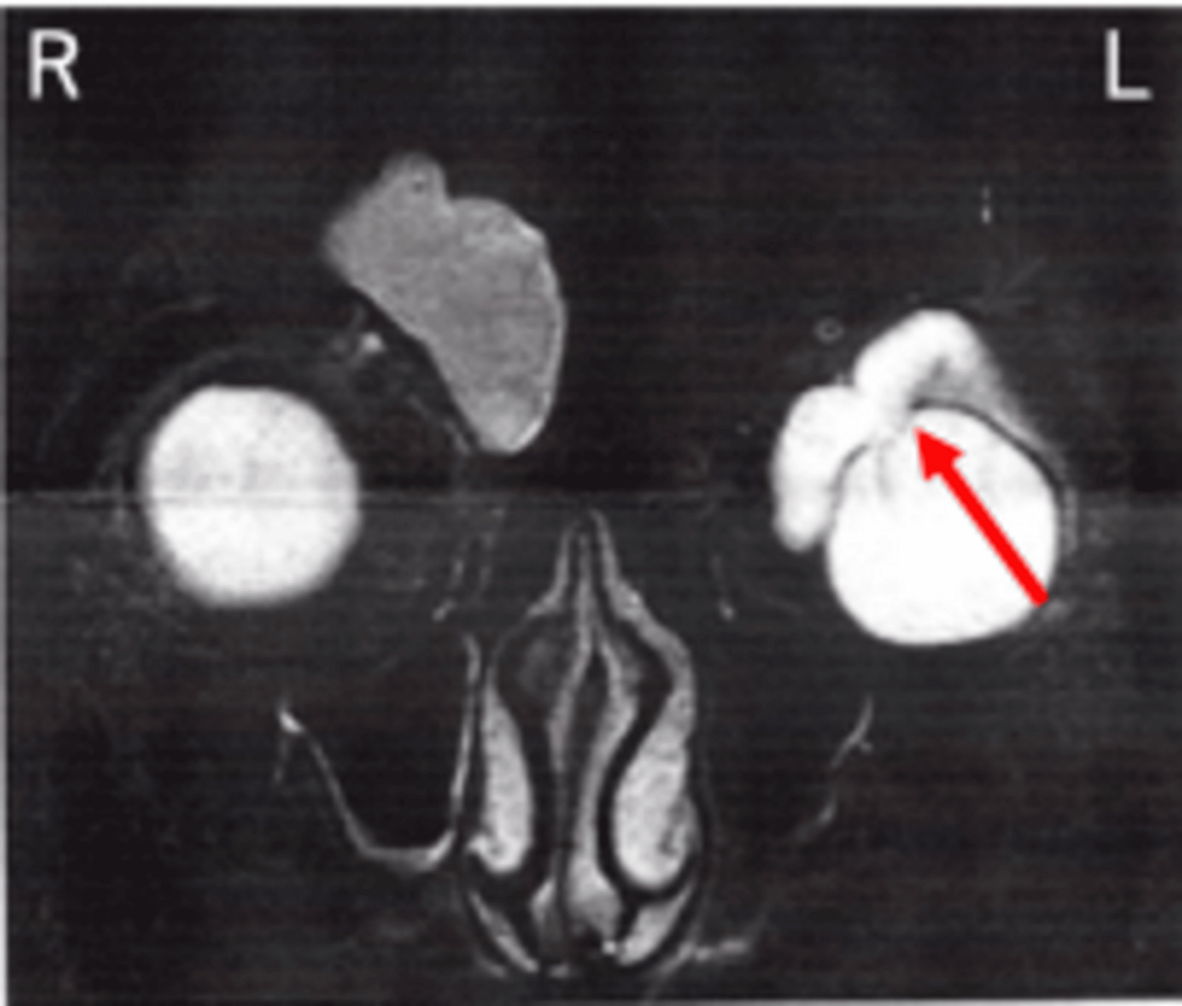
Magnetic resonance imaging findings. A high-signal area corresponding to the bulging part of the conjunctiva can be seen (red arrow).

MRI performed at the previous hospital showed a high-signal area with clear boundaries that corresponded to the bulging conjunctiva in the T2-weighted images (Figure 3).

Based on these findings, we concluded that the signs and symptoms were due to a swelling of the MIRAgel buckle. After receiving informed consent from the patient, we performed MIRAgel buckle removal surgery.

**Figure 4:**
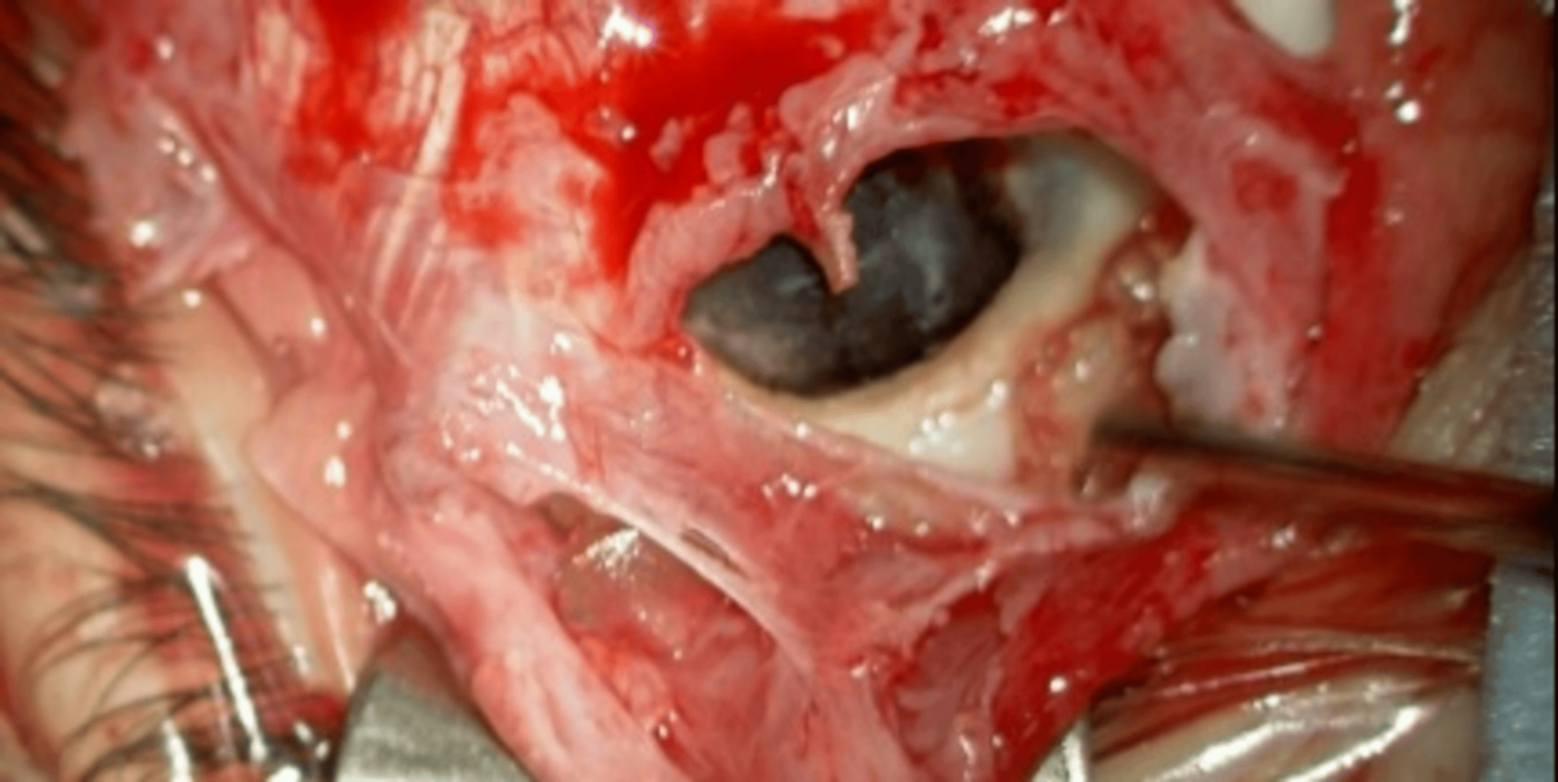
Intraoperative findings in the patient with an expanded MIRAgel buckle. The sclera where the MIRAgel buckle was placed cannot be seen, and the uvea is exposed.

During the surgery, the sclera beneath the MIRAgel buckle was not present and the uvea was exposed (Figure 4).

**Figure 5:**
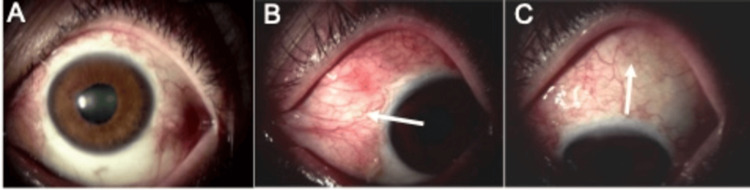
Postoperative slit-lamp images in the patient whose MIRAgel buckle was removed by suction. A: Frontal photograph. Mild cataracts remain; B: The bulging of the conjunctiva has improved (white arrow); C: The bulging of the conjunctiva has improved (white arrow).

It was then decided that the remaining fragments of the buckle needed to be removed without damaging the exposed uvea. A Rosen suction tube was used to remove the remaining MIRAgel buckle (Figure 5).

**Figure 6:**
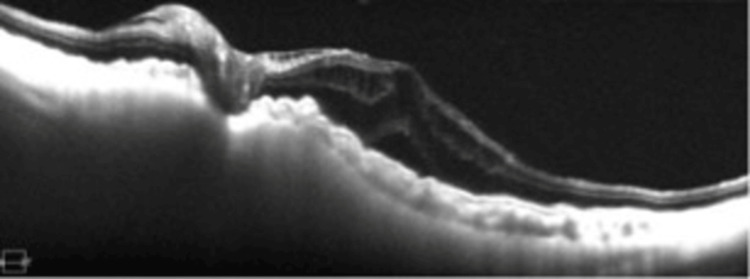
Postoperative OCT findings. The postoperative OCT image shows that the subretinal and intraretinal fluids are still present. OCT: Optical coherence tomography

Although the buckle was completely removed, the patient's postoperative BCVA was 2/200 and the IOP was 5 mmHg. In addition, we used steroid eye drops, but the macular edema remained and did not improve (Figure 6).

## Discussion

We present our findings in a case of necrotizing scleritis due to an expansion of an MIRAgel buckle implanted 30 years earlier. We suggest that when the MIRAgel begins to hydrolyze, it causes tissue damage due to inflammation caused by immune responses [6]. Our patient also had macular edema which was most likely due to the inflammation caused by a posterior scleritis-like condition caused by the expansion of the MIRAgel buckle [8]. The patient reported by Nakao et al. also had macular edema that persisted despite the removal of the MIRAgel buckle [7]. We suggest that the reason for this was that the sclera had degenerated, and the tissue damage caused by the hydrolysis of the MIRAgel had directly damaged the uvea causing severe intraocular inflammation. This inflammation was the cause of the macular edema, and removal of the MIRAgel buckle alone would not have improved the condition.

Then the reason for using a general-purpose suction tube for ophthalmic surgery to remove the MIRAgel in this case should be considered. The method of removing it using forceps or a strabismus hook reported in the past was not used because it was expected that it would be difficult to remove the fragmented MIRAgel completely, and there was a high possibility of damaging the uvea by the instruments [9]. A cryoprobe was not used either because there was a possibility of damaging the uvea and surrounding tissues due to freezing and coagulation [10].

The removal method using a Yunker suction tube reported by Nakao et al. was also considered to be very useful [7]. However in our patient, the exposure of the uvea was extensive, and delicate manipulation was required. Therefore, we decided that the surgical suction tube would be suitable because this tube is thin, is about 2mm, can be finely manipulated, and would be relatively safe.

In patients with MIRAgel implants, if symptoms such as bulging of the conjunctiva, conjunctival hyperemia, or ocular motility disorders are observed, it is necessary to consider the possibility of tissue damage due to MIRAgel expansion. Various removal methods have been proposed, but the method described in this report is simple and safe and is considered to be effective.

## Conclusions

We conclude that patients presenting with an expansion of a MIRAgel buckle accompanied by necrotizing-like scleritis can be successfully treated by removing the buckle with a surgical suction tube. In addition, we recommend that a MIRAgel buckle showing any signs of expansion should be removed because it can cause tissue damage due to hydrolysis.
